# Medical research: Are e-Sports really sports?

**DOI:** 10.1016/j.clinsp.2023.100190

**Published:** 2023-04-01

**Authors:** Fulvio A. Scorza, Ana C. Fiorini, Camila C. de Lima, Nilton Camilo, Eliana P. Magro, Liliane Guimarães, Claudio Pavanelli, Josef Finsterer, Antônio-Carlos G. de Almeida, Marcelo C.M. Fonseca, Reginado R. Fujita, Turibio L. de Barros, Miguel M.C. Scorza, Marcelo A. Moret, Lavínia Teixeira-Machado, Ricardo M. Arida

**Affiliations:** a Disciplina de Neurociência, Escola Paulista de Medicina/Universidade Federal de São Paulo (EPM/UNIFESP), São Paulo, SP, Brazil; b Escola Paulista de Medicina, Escola Paulista de Medicina/Universidade Federal de São Paulo (EPM/UNIFESP), São Paulo, SP, Brazil; c Departamento de Fonoaudiologia, Escola Paulista de Medicina/Universidade Federal de São Paulo (EPM/UNIFESP), São Paulo, SP, Brazil; d Programa de Estudos Pós-Graduado em Fonoaudiologia, Pontifícia Universidade Católica de São Paulo (PUC-SP), São Paulo, SP, Brazil; e The Villages Soccer Club, Florida, USA; f Neurology & Neurophysiology Center, Vienna, Austria; g Laboratório de Neurociência Experimental e Computacional, Departamento de Engenharia de Biossistemas, Universidade Federal de São João del-Rei (UFSJ), São João del-Rei, MG, Brazil; h Departamento de Ginecologia, Escola Paulista de Medicina/Universidade Federal de São Paulo (EPM/UNIFESP), São Paulo, SP, Brazil; i Departamento de Otorrinolaringologia, Escola Paulista de Medicina/Universidade Federal de São Paulo (EPM/UNIFESP), SP, São Paulo, Brazil; j Departamento de Fisiologia, Escola Paulista de Medicina/Universidade Federal de São Paulo (EPM/UNIFESP), São Paulo, SP, Brasil; k Physio Science, USA; l SENAI-Cimatec, Salvador, BA, Brazil; m Departamento de Ciências Exatas e da Terra (DCET), Universidade do Estado da Bahia (UNEB), Salvador, BA, Brazil; n Departamento de Educação em Saúde, Universidade Federal de Sergipe, São Cristovão, SE, Brazil

In the last decade, the Electronic Sports (eSports) industry has shown explosive growth, reaching a global audience of 557 million people in 2021.^1^^,^^2^ Several studies have projected that the eSports market should reach US$ 5.48 billion by 2029.^1^^,^^3^ However, there is no consensus on whether eSports should be considered a sport. Two questions come to the scene: what is sport and what is physical activity? In general, a sport can be defined as a game, competition, or activity that requires physical exertion and skill that contributes to physical conditioning, mental health, and social interaction.^1^^,^^4^ A new definition assumes that physical activity includes people that move, act, and occur in culturally specific spaces and contexts and are influenced by a unique array of interests, emotions, ideas, directions, and relationships.^5^ Recent studies confirm the great value of both sport (i.e., competitive, rule-based, structured activity) and physical activity in improving the psycho-social health and well-being of the human population.^6^ eSports enable viable careers and distinct categories, such as Sports (e.g., FIFA EA SPORTS), Firs-Person-Shooter (e.g., *Call of Duty*), Battle Royal (e.g., *Fortnite*), Strategy (e.g., *Starcraft*), and Multiplayer Online Battle Arena (e.g., *League of Legends*), and are supported by big companies to compete on an international level. eSports players who train several hours a day show similar characteristics as traditional sports athletes, such as interpersonal competition, the pursuit of athletic excellence, adherence to rules, achievement of goals, and inclusion of agility and coordination.^1^^,^^7^

It is extremely important to evaluate how eSports contribute to the improvement of health-related quality of life in the general population or in patients with chronic diseases. eSports activity may improve heart rate variability, endothelial function, blood pressure, lipid levels, and levels of inflammatory markers.^1^ But it should also be investigated whether a permanently high level of eSports could have adverse effects on the cardiovascular system or a therapeutic aid in controlling blood glucose management and overall health in individuals with diabetes and prediabetes. This can be used to assess whether eSports improves blood glucose levels, cardiovascular fitness, muscle strength, and insulin sensitivity and contributes to weight loss. A recent study showed that eSports players are significantly less active and have a higher body-fat percentage with lower lean body mass and bone mineral content when compared to non-eSports players.^8^

Studies have demonstrated the effectiveness of natural movement sounds, movement sonification, and rhythmic auditory information on athletic performance and motor (re)learning.^9^ It would be important to evaluate this aspect among eSports athletes, but also examine how the temporal information of repetitive acoustic stimuli is processed in the auditory cortex in these athletes and its relevance for the processing of speech sounds. The training of visual skills according to certain guidelines can lead to improved performance in various aspects of sports.^10^ Eyesight and eSports are an increasingly important topics among researchers as the nature of eSports raises important considerations for ocular health, and visual and perceptual functions, compared to traditional sports.^11^

The overexposure to blue light spectrum from LEDs can cause retinal and photoreceptor damage disrupts the circadian rhythm, and lower melatonin levels.^8^^,^^9^ There are few studies detailing the potential relationships between eSports and sleep behaviors. Significant individual differences in sleep among eSports players suggest that these athletes probably experience a high level of risk of sleep disorders compared to traditional athletes.^12^^,^^13^ Other studies have shown that better game performance is linked to better sleep and that poor performance can impact non-REM respiration rates and sleep.^14^ The routine and sleep profile of eSports players (e.g., quality of sleep, insomnia, and daytime sleepiness) should be analyzed. It makes sense to study in detail the visual sensory input, eye and hand co-ordination, eye dominance, ocular motility, vergence, peripheral awareness, and visual reaction time of eSports players.

Recent evidence demonstrated that physical exercise plays an important role in the regulation of the central nervous system promoting the release of molecules, involved in neuronal survival, differentiation, plasticity, and neurogenesis.^15^ Physical exercise has been shown to be a non-drug therapy for numerous neurological diseases and psychiatric diseases.^16^ Therefore, studies are needed to establish the neuroprotective repercussions of eSports.

Brazilian scientists explored the association between gender and leisure-time physical activity in a population-based sample of adults living in Brazil.^17^ The authors suggested the creation of intervention plans to increase the population level of leisure-time physical activity, depending on the gender of interest.^17^ Gender differences in physical activity levels have been demonstrated among children, adolescents, and adults, reporting males overall engage in higher levels of physical activity compared to females.^18^ There is a changeable culture emerging toward acceptance LGBTIQ+ people within society; sports being a dominant area of involvement for the LGBTIQ+ community.^19^

It will be also important to define if people who participate in eSports are more or less likely to develop a variety of types of cancer and to determine the specific dose of exercise that is optimal for primary cancer prevention or treatment. Then will be possible to determine if eSports activity is a useful adjunct to improve the deleterious sequelae (e.g., fatigue, muscle weakness, impaired functional capacity) that occur during cancer treatment.

Where do we go from here? First, the present research group totally agrees that eSports really are sports. The authors also believe that eSports offers multiple health benefits, promotes societal growth, and enables long-term prevention and treatment of chronic diseases while improving overall global health.^20^ It is essential to treat eSports athletes like any other athlete. Although American secondary education, colleges, and universities have begun to incorporate eSports into their traditional varsity sports programs,^21^ medical and translational research in eSports in academic and research institutions urgently needs to be improved. Finally, it should be noted that eSports is already and will continue to bring many economic benefits to various sectors of the economy, including education and health sectors.

## Conflicts of interest

The authors declare no conflicts of interest.
